# Recent Advances in the Recombinant Biosynthesis of Polyphenols

**DOI:** 10.3389/fmicb.2017.02259

**Published:** 2017-11-16

**Authors:** Sonam Chouhan, Kanika Sharma, Jian Zha, Sanjay Guleria, Mattheos A. G. Koffas

**Affiliations:** ^1^Natural Product Laboratory, Division of Biochemistry, Faculty of Basic Sciences, Sher-e-Kashmir University of Agricultural Sciences and Technology of Jammu, Jammu, India; ^2^Department of Chemical and Biological Engineering, Center for Biotechnology and Interdisciplinary Studies, Rensselaer Polytechnic Institute, Troy, NY, United States; ^3^Department of Biological Sciences, Center for Biotechnology and Interdisciplinary Studies, Rensselaer Polytechnic Institute, Troy, NY, United States

**Keywords:** flavonoids, anthocyanins, curcuminoids, stilbenes, polyphenols, phytochemicals

## Abstract

Plants are the source of various natural compounds with pharmaceutical and nutraceutical importance which have shown numerous health benefits with relatively fewer side effects. However, extraction of these compounds from native producers cannot meet the ever-increasing demands of the growing population due to, among other things, the limited production of the active compound(s). Their production depends upon the metabolic demands of the plant and is also subjected to environmental conditions, abundance of crop species and seasonal variations. Moreover, their extraction from plants requires complex downstream processing and can also lead to the extinction of many useful plant varieties. Microbial engineering is one of the alternative approaches which can meet the global demand for natural products in an eco-friendly manner. Metabolic engineering of microbes or pathway reconstruction using synthetic biology tools and novel enzymes lead to the generation of a diversity of compounds (like flavonoids, stilbenes, anthocyanins etc.) and their natural and non-natural derivatives. Strain and pathway optimization, pathway regulation and tolerance engineering have produced microbial cell factories into which the metabolic pathway of plants can be introduced for the production of compounds of interest on an industrial scale in an economical and eco-friendly way. While microbial production of phytochemicals needs to further increase product titer if it is ever to become a commercial success. The present review covers the advancements made for the improvement of microbial cell factories in order to increase the product titer of recombinant polyphenolic compounds.

## Introduction

Natural products that are produced as a result of plant metabolism, commonly referred to as phytochemicals, represent an enormous repository of bioactive compounds having pharmaceutical and biotechnological importance. Mankind has long exploited the impressive synthetic capacity of plants as a source of flavors, colorants, fragrances, pharmaceutical drugs and traditional medicines(Mora-Pale et al., 2013). The products of plant origin continue to play a leading role in drug discovery due to their specialized skeletal structures and functional groups (Bourgaud et al., 2001; Newman and Cragg, 2007; Li and Vederas, 2009). Currently, commercialized phytochemicals are still manufactured by extraction from their native plant sources or by semi-synthesis from extracted intermediates of end products (Chemler and Koffas, 2008). Low toxicity displayed by many phytochemicals and better success rates of natural products entering into a phase I testing (25 versus 6% of conventional chemicals) are the major advantages of using these compounds in human applications (Schmid, 2003). However, low yield and the complicated downstream purification processes are the major bottlenecks in the plant extraction process (Chemler et al., 2006; Wang et al., 2016).

An array of natural products, including several phytochemicals, have been elegantly synthesized/modified by organic chemists but such processes of chemical synthesis are usually overshadowed by inherent disadvantages such as expensive precursors, use of toxic catalysts and extreme reaction conditions that make them not amenable to large scale production (Chemler and Koffas, 2008). In addition, structural complexity, presence of chiral centers and labile connectivities present in many of these compounds make their chemical synthesis difficult. Thus, an alternative approach in the production and development of such compounds is microbial biosynthesis (Demain and Adrio, 2008). In order to meet the industrial application requirements, the three pillars of metabolic engineering, namely titer, yield and rate, need to be improved to levels that make recombinant microorganisms competitive against current production methods (Nielsen, 2001).

Metabolic engineering is defined as the introduction of rational changes in the genetic makeup of an organism for improving its phenotype such as improving capabilities of biosynthesis (Stephanopoulos et al., 1998; Nielsen, 2001). Protein engineering, another engineering field commonly applied in improving microbial production of phytochemicals, focuses on improvements in the catalytic efficiency of enzymes as well as diversifying enzyme promiscuity (Savile et al., 2010). For the synthesis of phytochemicals, two main objectives toward which metabolic and protein engineering have been applied are: the increase of the yield of the target compound and modification of the scaffold of natural product for improved properties. Synthetic biology approaches have also been applied in improving phytochemical production titers, mainly by developing novel sensor systems that can be applied for dynamic control of metabolic fluxes and for high-throughput screening (Greber and Fussenegger, 2007; Keasling, 2008; Weber and Fussenegger, 2009).

Flavonoids belong to the phenolic family of compounds in which a linear carbon chain join two benzene rings (Lim and Koffas, 2010). These are produced against biotic and abiotic stresses of various types like microbial invasions, environmental stresses, physical injury etc. (Treutter, 2006). Flavonoids have proven to have many important health benefits for humans (Williams et al., 2004).

Anthocyanins belong to the flavonoid group of polyphenols and are important chemicals in the plant kingdom, serving various roles mainly due to their color. In human health, interest in anthocyanins stems from their antioxidant properties (Tsuda, 2012).

Curcuminoids are polyphenolic compounds found as active ingredients in the dietary spice turmeric (*Curcuma longa*) where the major component present is curcumin. Due to the several therapeutic properties of curcumin (antioxidant, anti-Parkinson, anti-inflammatory, anti- HIV and anticancer), it has been used in traditional medicine and as a food additive (Reddy et al., 2005; Goel et al., 2008; Aggarwal and Harikumar, 2009; Prasad et al., 2014; Sarkar et al., 2016). In this review, we have covered the recent advances related to engineering the production of polyphenolic compounds, specifically flavonoids, anthocyanins and curcuminoids, in recombinant microorganisms. Production titers of certain polyphenols through metabolic engineering in micro-organisms are presented in **Table 1**.

**Table 1:** Production titers of certain polyphenols through metabolic engineering in micro-organisms.

Micro-organism	Biosynthetic components	End-product	Titer	Reference
*Saccharomyces cerevisiae*	PAL (*Rhodosporidium toruloides*), 4CL (*Arabidopsis thaliana*), CHS (*Hypericum androsaemum*)	Naringenin	7 mg/L	Jiang et al., 2005
*Escherichia coli*	PAL (*Rhodotorula rubra*), 4CL (S*treptomyces coelicolor*), CHS (*Glycyrrhiza echinata*), CHI (*Pureria lobata*), ACC (*Cornybacterium glutamicum*)	Naringenin	57 mg/L	Miyahisa et al., 2005
*Escherichia coli*	F3H (*Malus domestica*), ANS (*Malus domestica*), DFR (*Anthurium andraeanum)*, F3GT (*Petunia hybrida)*	Cyanidin 3-*O*-glucoside	6 μg/ L	Yan et al., 2005
*Escherichia coli*	F3H (*Malus domestica*), ANS (*Malus domestica*), DFR (*Anthurium andraeanum)*, F3GT (*Petunia hybrida)*	Pelargonidin 3-*O*-glucoside	5.6 μg/L	Yan et al., 2005
*Saccharomyces cerevisae*	C4H (*Arabidopsis thaliana*), 4CL (*Petroselinum. crismum*), CHS, CHI (*Petunia x hybrida*)	Naringenin	28.3 mg/L	Yan et al., 2005
*Escherichia coli*	4CL (*Lithospermum erythrorhizon*), CHS, CHI (*Glyccyrrhiza echinata*), STS (*Arachis hypogaea*), FNS (*Petroselinum crismum*), F3H, FLS (Citrus), ACC (*Cornybacterium glutamicum*).	Flavonols	33 mg/L	Katsuyama et al., 2007
*Escherichia coli*	4CL (*Lithospermum erythrorhizon*), CHS, CHI (*Glycyrrhiza echinata*), STS (*Arachis hypogaea*), FNS (*Petroselinum crismum*), F3H, FLS (Citrus), ACC (*Cornybacterium glutamicum*).	Flavanones	102 mg/L	Katsuyama et al., 2007
*Escherichia coli*	PAL (*Rhodotorula rubra*), 4CL (*L. ithospermum erythrorhizon*), CUS (Oryza sativa).	Bisdemethoxy curcumin	53.4 mg/L	Katsuyama et al., 2008
*Escherichia coli*	PAL (*R. rubra*), 4CL (*L. ithospermum erythrorhizon*), CUS (*Oryza sativa*).	Curcumin	113 mg/L	Katsuyama et al., 2008
*Saccharomyces cerevisiae*	PAL (*Populus trichocarpa*), C4H (*Glycine max*), 4CL(*Glycine max*), IFS, CHS, CHI ((*Glycine max*)	Genistein	7.7 mg/L	Trantas et al., 2009
*Escherichia coli*	4CL (P*etroselinum* crismum), CHS (*Petunia x hybrida*), CHI (*Medicago sativa*), ACC (*Photorhabdus luminescens*), PGK, PDH (*Escherichia coli*)	Naringenin	474 mg/L	Xu et al., 2011
*Saccharomyces cerevisiae*	PAL, C4H, CPR, 4CL, CHS, CHI (*Arabidopsis thaliana*), TAL (*Rhodobacter capsulatus*), ARO4^G2265^ (*Saccharomyces cerevisiae*)	Naringenin	109 mg/L	Koopman et al., 2012
*Escherichia coli*	4CL (*P. crispum*), CHS (*P. hybrid*), CHI (*M. sativa*)	7-*O*-methyl aromadendrin	30 mg/L	Malla et al., 2012
*Escherichia coli*	PAL (*Rhodotorula glutinis*), 4CL (*Petroselinum crispum*), CHS (*Petunia hybrid*), CHI (*Medicago sativa*)	Pinocembrin	40 mg/L	Wu et al., 2013a
*Escherichia coli*	PAL (*Rhodotorula glutinis*), 4CL (*Petroselinum crispum*), CHS (*Petunia hybrid*), CHI (*Medicago sativa*), F3’H (*Gerbera hybrida*), CPR (*Catharanthus roseus*)	Eriodictyol	107 mg/L	Zhu et al., 2014
*Escherichia coli*	DCS (*Curcuma longa)*, CURS1 (*Curcuma longa)*, 4CL1 (*Arabidopsis thaliana*)	Curcumin	70 mg/ml	Rodrigues et al., 2015
*Escherichia coli*	ANS (*Malus domestica*), F3GT (*Petunia hybrida)*	Cyanidin 3-*O-*glucoside	350 mg/L	Lim et al., 2015
*Escherichia coli*	PAL (*Rhodotorula glutinis*), 4CL (*Petroselinum crispum*), CHS (*Petunia hybrid*), CHI (*Medicago sativa*)	Pinocembrin	525.8 mg/L	Wu et al., 2016
*Escherichia coli*	DCS, CURS1 (*Curcuma longa)*, 4CL1 (*Arabidopsis thaliana*)	Curcumin	17 μM	Joana et al., 2017
*Escherichia coli*	*PAL* (*A. thaliana*), *TAL* (*Saccharothrix espanaensis*), *4CL* (*Oryza sativa*), *CUS* (*O. sativa*)	Curcuminoids	6.95 mg/L	Kim et al., 2017


## Flavonoids

**FIGURE 1:**
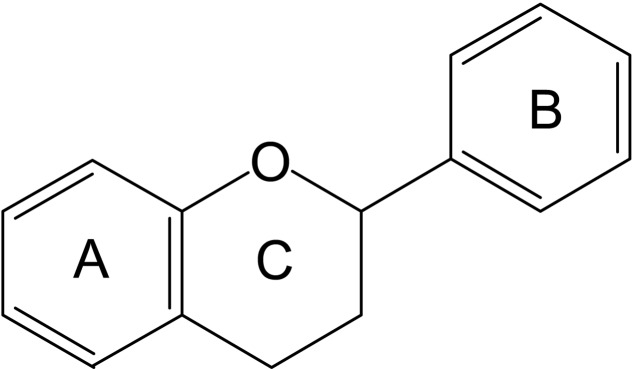
Flavonoid core structure.

Flavonoids are members of the polyphenols family of phytochemicals. The general structural formula of these compounds is C6–C3–C6. A heterocyclic ring joins the two C6 units (Ring A and Ring B) forming a 15-carbon phenylpropanoid core (**Figure 1**).

**FIGURE 2:**
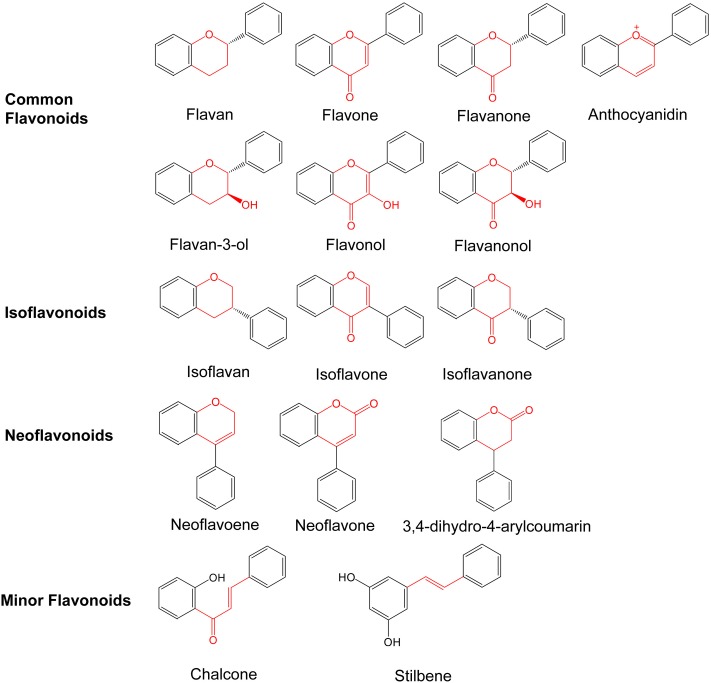
Flavonoid skeletons and ring designations.

The main differences between flavonoids relate to the patterns of hydroxylation, the second aromatic ring position and the heterocyclic ring saturation (Grotewold, 2006). Ring C is variously modified by methylation, methoxylation, alkylation, oxidation, *C*- and *O*-glycosylation, rearrangement, and hydroxylation, chemical modifications which lead to the formation of more than 9,000 flavonoid derivatives. These compounds have many important properties, including antioxidant, antibacterial, antiviral, and anti-cancer (Leonard et al., 2008). Flavonoids can be divided into three major classes based on the position of the linkage of ring B to the ring C: common flavonoids, isoflavonoids, and neoflavonids (Pandey et al., 2016) (**Figure 2**). According to the modifications to the ring C (dehydrogenation of C2, hydroxylation at C3 or C4, and oxidation at C4), the common flavonoids contain several subclasses, including flavan, flavone, flavanone, flavanonol, flavanol, flavonol, and anthocyanin. Fruits are a rich source of flavonols, particularly the skin of grapes and apples (Tsao et al., 2003). Monomeric flavonols like catechins and epicatechins are present in tea-leaves and cacao beans as the major flavonoids (Prior et al., 2001; Si et al., 2006). Anthocyanins form another large subgroup of flavonoid which are the principal components of the red, blue and purple pigments of the majority of flower petals, fruits and vegetables. Anthocyanins commonly refer to the glycosidic forms of these compounds in plants. Based on the hydroxylation and methoxylation pattern on ring B, and the glycosylation with different sugar units, more than 500 anthocyanins have been identified (Tsao, 2010). In isoflavonoids, such as geninstein, the C3 position of Ring C is attached to Ring B. These are mostly present in the leguminous family. Neoflavonids consist of three subclasses. Other compounds having a similar skeleton are sorted into the subclass of minor flavonoids, such as chalcone and stilbene.

### Biosynthesis of Flavonoids in Plants

**FIGURE 3:**
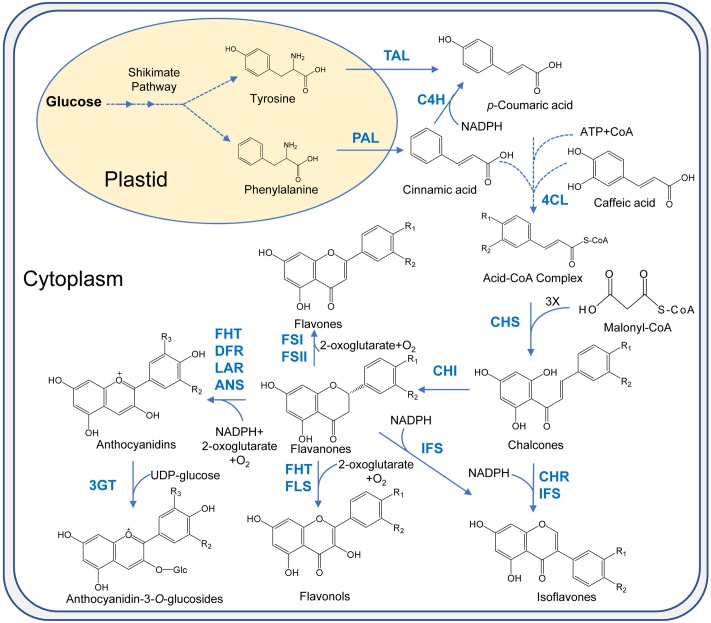
Flavonoids biosynthesis pathway in plants. PAL, phenyl ammonia lyase; TAL, tyrosine ammonia lyase; C4H, cinnamate 4-hydroxylase; 4CL, 4-coumarate: CoA ligase; CHS, chalcone synthase; CHI, chalcone synthase; IFS, isoflavone synthase; FSI, soluble flavone synthase; FSII, membrane-bound flavone synthase; FHT, flavanone 3β-hydroxylase; FLS, flavonol synthase; IFS, isoflavone synthase; CHR, chalcone reductase; DFR, dihydroflavonol 4-reductase; LAR, leucocyanidin reductase; ANS, anthocyanidin synthase; 3GT, flavonoid 3-*O*-glycosyltransferase.

Complexes of various enzymes for flavonoid biosynthesis are present on the cytosolic face of endoplasmic reticulum membranes which carry out flavonoid biosynthesis. Flavonoid biosynthesis branches off the phenylpropanoid pathway. It starts with the deamination reaction in which amino acid phenylalanine is converted to cinnamic acid by phenylalanine ammonia lyase (PAL) (**Figure 3**). Then, cinnamate-4-hydroxylase (C4H) carries out the oxidation of cinnamic acid to 4-coumaric acid. In the next step, the synthesis of 4-coumaroyl-CoA is catalyzed by 4-coumarate: CoA ligase (4CL). Subsequently, three molecules of malonyl-CoA are condensed with one molecule of CoA ester by chalcone synthase (CHS) to form chalcone. More than 9,000 flavonoids are obtained from chalcone by various enzymes like isomerases, hydroxylases, oxido-reductases as well as post modification enzymes like glycosyltransferases, methyltransferases, acyltransferases (Veitch and Grayer, 2011; Iwashina, 2015). Downstream flavonoids are obtained from (2*S*)-flavanones (precursor flavonoid) which are obtained by the stereospecific isomerization of chalcones in the reaction catalyzed by chalcone isomerase (CHI). Hydroxylation of (2*S*)-flavanones at the 3-carbon position by flavanone 3β-hydroxylase (FHT) give rise to dihydroflavanols whose reduction is then catalyzed by dihydroflavonol 4-reductase (DFR) at 4-carbon position to produce leucoanthocyanidins. These molecules are unstable and get reduced by leucoanthocyanidin reductase (LAR) to flavan-3-ols or catechins. Anthocyanidins are synthesized from leucoanthocyanidins and flavan-3-ols by the enzyme anthocyanidin synthase (ANS) which are then glycosylated at the 3-carbon by the enzyme UDP-glucose:flavonoid 3-*O*-glucosyltransferase (3GT), yielding anthocyanins (Davies and Schwinn, 2005). The structural diversity and related structures including isoflavonoids, condensed tannins, aurones, and stilbenes are generated by the action of enzymes which catalyze the addition of functional groups. Various biologically active properties of flavonoids are due to this functionalization.

The increasing demands for flavonoids and other secondary metabolites cannot be satisfied by their extraction solely from plants because plants produce several of these compounds only in limited amounts and also only under some specific environmental conditions or certain types of biotic or abiotic stresses. Other factors responsible for low yield of flavonoid extraction from plants include seasonal variations, naturally low levels of the metabolites of interest in the plant due to the production of large number of byproducts and their strict metabolic regulation, the complex nature of flavonoids as well as the requirement for complex extraction procedures involving toxic chemicals and complicated downstream processing (Paterson and Anderson, 2005; Matkowski, 2008; Keasling, 2010). Moreover, it is difficult to cultivate many plants containing high-value compounds or over harvesting may result in their depletion. Therefore, alternative production methods are needed for the large scale production of flavonoids (Fowler et al., 2009; Wang et al., 2016). Chemical synthesis is one of the alternative routes but it is difficult to scale up and cannot easily perform important modifications, such as targeted hydroxylations and glycosylations.

Another approach to increase product yield is the metabolic engineering of plants, however, the complexity of plant cells, their muticellular make up and the complex and strict biosynthetic regulation create difficulties in this approach. The use of plant cell cultures for production purposes is another production method, and taxol, ginseng and anthocyanins have been produced using this approach. There are various limiting factors in this approach such as culture heterogeneity, variability in yields, low growth rates, unstable cultures, susceptibility to stresses and aggregation (Wilson and Roberts, 2012). However, in many cases elication can be used for increasing the production of secondary metabolites by using elicitors such as methyljasmonate, salicylic acid, chitosan and metal ions and plant culture systems such as cell suspension, hairy roots and adventitious roots (Karla et al., 2016).

Various natural as well as novel flavonoid derivatives can be synthesized in microbial cell factories by following metabolic engineering, synthetic biology and protein engineering approaches (Kolewe et al., 2008). There are many benefits in using recombinant microbes for flavonoid production. For example this strategy has the potential to be more economical as it can effectively convert cheap carbon sources such as glycerol and cellulose to high-value chemicals, ecofriendly, requires less time, has easy downstream processing and also decreases the loss of pathway intermediates to competing pathways often present in the natural host. These cell factories have also the potential to produce novel flavonoid derivatives which might have more pharmaceutical and nutraceutical value. Moreover microbes can be easily grown, have fast growth rates and also various metabolic engineering tools are available for carrying out elaborate engineering of the strains. (Hwang et al., 2003; Yan et al., 2005, 2007; Leonard and Koffas, 2007; Wu et al., 2013a).

### Metabolic Engineering of Flavonoid Biosynthesis in Microbes

The most commonly used prokaryotic and eukaryotic organisms for metabolic engineering are the bacterium *Escherichia coli* and the yeast *Saccharomyces cerevisiae*, respectively. Both of these organisms are very well-characterized, easy to manipulate and easy to grow especially in scaled-up fermenters. In recent years, various novel biosynthetic pathways as well as metabolic engineering tools have been developed and applied that allow reconstruction of complex pathways for production of flavonoids in these two microorganisms.

Metabolic engineering of microbes for flavonoid production requires selection and optimization of host strain, determination of targets for gene manipulations and knowledge of the enzymes involved in the biosynthetic pathways. In general, metabolic engineering of natural product biosynthesis in microbes consists of the following steps: bioprospecting and recombinant pathway design (recombineering); selection and cloning or construction of heterologous genes; production host choice, vector choice, and transformation of heterologous genes into host; optimizing the expression, folding, and activity of plant proteins in the microbial hosts (often via protein engineering); strain improvement via carbon flux redistribution, toxicity reduction, transporter engineering, removal of regulatory restrictions, enzyme colocalization or compartmentalization and pathway balancing (Cress et al., 2015; Jones et al., 2015; He et al., 2017). Although the whole procedure for metabolic engineering is standardized and conceptualized, many regulatory control mechanisms in nature are not fully understood, and therefore, systematic and informatics-based approaches combining genomic, proteomic, and metabolomic analyses have been utilized (Vemuri and Aristidou, 2005).

Gene orthologs from different plant sources can be used to construct efficient metabolic pathways (Lee et al., 2012). Using flux balance analysis, computational approaches can be applied for the prediction of genetic perturbation targets that would channel more carbon flux toward target chemicals (Mederma et al., 2012). New catalytic functions can also be engineered by site directed mutagenesis and directed evolution (Wang et al., 2011b; Lee et al., 2012). Despite exceptional advances in the fields of metabolic engineering and synthetic biology, the synthesis of flavonoids by microbial engineering has only been demonstrated at the laboratory scale (Leonard et al., 2008; Wu et al., 2013a). There are various factors which limit the large scale production of flavonoids using recombinant microorganisms. One of the most important factors is the low intracellular concentration of malonyl CoA, the precursor metabolite for the biosynthesis of flavonoids. Other factors are the low availability of aromatic amino acids tyrosine and phenylalanine (the two immediate precursors of phenylpropanoic acids), the interconnectivity of cellular metabolism that results in unpredictable phenotypes, the poor expression of some of the enzymes involved in the metabolic pathways and the instability and/or low solubility of some flavonoids, such as the anthocyanins. New perspectives on the optimization of production strain and process are offered by the emergence of systems metabolic engineering, which is a combination of systems biology with synthetic biology and evolutionary engineering at the systems level (Sagt, 2013).

The early work in the area of recombinant production of flavonoids relied on feeding with phenylpropanoic acid precursors, in order to uncouple the production of flavonoids from the availability of tyrosine and phenylalanine. However, in the past 10 years, many cost-effective methods have been developed by which microbial synthesis of naringenin and pinocembrin can be achieved without the addition of any costly precursor molecules. For this purpose, four enzymes, i.e., phenylalanine/tyrosine ammonia lyase (PAL/TAL), 4-coumarate:CoA ligase (4CL), chalcone synthase (CHS), and chalcone isomerase (CHI) were assembled into a gene construct and introduced either into *E. coli* (Santos et al., 2011; Wu et al., 2013a) or *S. cerevisiae* (Koopman et al., 2012). This resulted in the production of 29 mg/L and 40 mg/L of naringenin and pinocembrin respectively from glucose in *E. coli* strains (Santos et al., 2011; Wu et al., 2013a) whereas naringenin production from glucose in *S. cerevisiae* was 109 mg/L (Koopman et al., 2012). Meanwhile, kaempferol and quercetin have been produced using *p*-coumaric acid as precursor and fisetin was produced using L-tyrosine as a precursor (Leonard et al., 2006a,b; Santos et al., 2011; Stahlhut et al., 2015).

*Escherichia coli* has been used for the production of many flavonoids since 2003 (Hwang et al., 2003; Miyahisa et al., 2005; Leonard et al., 2007, 2008). The production of plant-derived flavonoids in *E. coli* was first reported by Hwang et al. (2003) Three enzymes from different sources were used to engineer a recombinant *E. coli* strain namely phenylalanine ammonia lyase (PAL) from *Rhodotorula rubra*, coumarate: coenzyme A ligase (4CL) from *Streptomyces coelicolor*, and chalcone synthase (CHS) from *Glycyrrhiza echinata.* Naringenin chalcone and pinocembrin chalcone were produced by feeding the engineered *E. coli* strain with tyrosine and phenylalanine respectively. This strategy involved the use of three different vectors in which the numbers of T7 promoter and ribosome binding sequences (RBS) were varied: only one T7 promoter and one ribosome-binding sequence (RBS) controlled all the genes encoding for PAL, 4CL, and CHS in the first vector; in the second vector the three genes were under the control of the T7 promoter with RBS at appropriate positions; whereas in the third construct each gene was preceded by T7 promoters and RBS sites. The highest production of naringenin was obtained using the last vector, with 0.45 mg/L of naringenin produced. In 2005, Miyahisa and coworkers combined PAL, CHS and 4CL with chalcone isomerase (CHI) in a vector for the optimization of gene expression. The production titer of naringenin using this construct was increased to 60 mg/L. At the same time, Yan et al. (2005) constructed and introduced a four-step flavanone biosynthetic pathway into *S. cerevisiae*. Flavanones naringenin and pinocembrin were produced 62 and 22 times more efficiently by the recombinant yeast strain upon feeding with phenylpropanoid acids than the previously reported recombinant prokaryotic strains (Yan et al., 2005).

Many flavonoids such as genistein, kaempferol, and quercetin were produced by feeding naringenin to engineered yeast cells (Trantas et al., 2009). Koopman and colleagues also used an engineered yeast strain and obtained 108.90 mg/L naringenin (Koopman et al., 2012). As already mentioned, a limiting factor in the microbial production of flavonoids is the low intracellular concentration of malonyl CoA (Wang et al., 2011b). Various novel strategies have been employed to enhance the intracellular pool of this important molecule. Perhaps the most popular one included the engineering of assimilation pathways of malonate (Leonard et al., 2007, 2008) and acetate and also the overexpression of acetyl-CoA carboxylase, ACC (Miyahisa et al., 2005). The production of naringenin from tyrosine and pinocembrin from phenylalanine was increased up to three and four fold respectively by the overexpression of enzyme ACC. In another approach for improving the intracellular concentration of malonyl CoA, both ACC and BPL (biotin ligase encoded by birA) were overexpressed simultaneously as the carboxylase domain of ACC becomes functional by biotinylation which takes place through the action of biotin ligase. For this purpose, genes encoding for ACC and BPL from different sources were combined so as to optimize the combination which included the BPL from *E. coli*, *Photorhabdus luminescens* and a chimeric BPL. Using this approach, pinocembrin and naringenin production was increased upto 367 mg/L and 69 mg/L respectively by co-expressing both ACC and BPL from *P. luminescens* (Leonard et al., 2007). The malonyl CoA pool can also be enriched by providing exogenous sources of acetate that improves the production of the flavanone pinocembrin to a final titer of 429 mg/L (Leonard et al., 2007). In another study conducted by Leonard et al. (2008), two strategies were employed for improving the intracellular availability of malonyl CoA. The first method involved the simultaneous overexpression of flavanone biosynthetic genes and the genes for recombinant malonate assimilation pathway from *Rhizobium trifolii* (MatB and MatC) which transports exogenously supplemented malonate and then converts it into malonyl CoA. This method resulted in titers of 480 mg/L and 155 mg/L of pinocembrin and naringenin respectively. In the second strategy, fatty acid pathway inhibitor cerulenin was used to inhibit two fatty acid biosynthetic enzymes, FabB and FabF in an effort to reduce the amount of malonyl CoA lost to the synthesis of fatty acids. The dose of cerulenin needed to be optimized for the optimization of product titers. 0.2 mM of cerulenin led to maximum pinocembrin yield of 710 mg/L (Leonard et al., 2008).

Leonard et al. (2005) used two isoforms of the flavones synthase (FS) enzyme (FSI is soluble and FS II is membrane bound) for engineering the yeast strains and produced various flavones (chrysin, apigenin) and also intermediate flavanones (eriodictyol, naringenin, pinocembrin) using phenylpropanoid precursors. *E. coli* strains, expressing five plant genes for flavone production were also engineered with the flavone synthase (FSI) derived from parsley which causes production of genkwanin, luteolin and apigenin in appreciable amounts after 24 h culture (Leonard et al., 2005).

In addition to pathway engineering, codon optimization, enzyme engineering and mutasynthesis can be used to increase production, and to produce novel compounds in microbial cell factories. The catalytic power of enzymes of the flavonoid pathway can be optimized by various methods like site directed mutagenesis, creation of enzymes with desired functions, formation of fusion proteins, choosing or screening of efficient enzymes from different plant sources, enzyme engineering or directed evolution (Wang et al., 2012; Tee and Wong, 2013). Phenylalanine ammoia lyase (PAL) and tyrosine ammonia lyase (TAL) are the main enzymes in the pathway leading to flavonoid synthesis. These enzymes appear to be the rate limiting steps in the metabolic pathway leading to the biosynthesis of flavanones (Lim et al., 2011; Santos et al., 2011). In order to alleviate these bottlenecks, Wang et al. (2011a) resynthesised TAL with desired functions by codon optimization of the bacterial enzyme in yeast. The efficiency of translation was greatly enhanced, thereby resulting in increased production of *p*-coumaric acid, an intermediate in the flavonoid synthesis pathway. Wu et al. (2013a) also used codon optimization for increasing the expression, in *E. coli*, of the enzymes- PAL/TAL, chalcone synthase (CHS), chalcone isomerase (CHI) and 4-coumarate: CoA ligase (4CL) thus increasing the production titer of resveratrol and pinocembrin to 35 mg/L and 40 mg/L respectively. In addition, the expression of stilbene synthase (STS) was also increased by codon optimization (Wu et al., 2013b). Bhan et al. (2015) exploited the thioesterase-like property of STSs (belonging to type III polyketide synthase superfamily) by site dissected mutagenesis to further diversify the chemical space of aromatic polyketides. By comparing the randomness in the substrate binding properties of the wild type enzyme and all mutants using unnatural substrates, 15 novel aromatic polyketide molecules were produced. Another strategy of increasing enzyme efficiency is the formation of fusion proteins in order to mimic the protein–protein interactions that naturally occur among flavonoid biosynthetic enzymes in plants. Yan et al. (2008) produced two different translationally fused proteins (differing in the order of placement of the genes), specifically an *At3GT* derived from *Arabidopsis thaliana* and a *PhANS* derived from *Petunia hybrida.* Both of these enzymes catalyze the formation of glycosylated anthocyanins from precursor flavan-3-ols and leucoanthocyanidins. Using this approach, the production of cyanidin 3-*O*-glucoside was increased by 17% (45.5 mg/L) when *At3GT* was fused at the N-terminus of *PhANS.*

Another method of increasing enzyme expression is the optimization of Ribosome Binding Site (RBS) and promoter strength. Modification of promoter and ribosome binding site strength have been used in order to optimize flavonoid biosynthesis in recombinant hosts. Application of promoter engineering in flavonoid biosynthesis pathway in *E. coli* hosts made possible the synthesis of 100.64 mg/L of naringenin from glucose, 2.3 g/L of resveratrol from *p*-coumaric acid (Lim et al., 2011), 40.02 mg/L of (*2S*)-pinocembrin from glucose (Wu et al., 2013a) and 107 mg/L of eriodictyol from L-tyrosine (Zhu et al., 2014). Translation efficiency is directly affected by varying the RBS sequences as these mediate translation initiation (Boyle and Silver, 2012). Na et al., 2013 developed an *E. coli* strain for the efficient production of tyrosine by optimization of the ribosome binding sites (RBS) of *tyrR*, *csrA*, *pgi* and *ppc* genes. As a result, increased production of L-tyrosine (2 g/L), an alternative precursor for flavonoid biosynthesis, was achieved. Umar et al., 2012 engineered the gene constructs of (-) epicatechin, (-) epicatechin gallate, (+) epicatechin hydrate, and (-) catechin gallate by using different RBSs resulting in the production of 0.01 mg/L, 0.36 mg/L, 0.13 mg/L and 0.04 mg/L of the respective compounds.

Flavonoid production in heterologous hosts can also be increased by pathway balancing, substrate channeling using synthetic scaffolds and transporters, preventing intracellular accumulation of byproducts, and tolerance engineering of the host stain. Microbial cells have a well-defined feedback regulation system and hence increased intracellular accumulation of the desired metabolite leads to feedback inhibition of the concerned pathway. To reduce the intracellular accumulation of the pathway end product, specific transporters can be used which pump out the final product into the extracellular space thus reducing intracellular concentration of flavonoids and hence feedback inhibition. Within this context, improvement of resveratrol production was obtained by using high capacity bacterial AraE transporters because its chemical structure resembles that of flavonoid scaffolds (Wang et al., 2011a). Transporters can also increase the intracellular concentration of precursor or substrate molecules by pumping them into the microbial cells so as to increase the pathway flux toward the desired product (Lee et al., 2012).

Another bottleneck is the inhibition of desired molecules production due to the accumulation of unwanted byproducts. To overcome this, the genes encoding enzymes of competing pathways can be downregulated by gene deletion and the genes of the desired pathway can be amplified or upregulated (Yadav et al., 2012). Koopman et al. (2012) increased the concentration of aromatic amino acids (the flavonoid precursors) by reducing the formation of the byproduct phenylpyruvate decarboxylase by feedback inhibition of the enzyme 3-deoxy-D-arabinose-heptulosonate-7-phosphate synthase.

Synthetic scaffolds also increase the efficiency of the pathway by channeling the substrate and pathway intermediates and thus minimizing the diffusion of reactive intermediates (Moon et al., 2010). These scaffolds are constructed by assembling different enzymes needed for a specific reaction through non–covalent interactions like the naturally present organization of functionally related enzymes. These scaffolds maintain the stability within the host cell during reaction conditions. The yield of resveratrol was increased upto five fold by using synthetic scaffold strategy in comparison to the control (Wang and Yu, 2011). Similar scaffolds and artificial enzyme complexes were explored in a three-step recombinant pathway comprised of F3H, DFR and LAR for the conversion of flavanones to flavan-3-ols (Zhao et al., 2015).

Microbial cell factories can be optimized for the production of desired compounds by using various synthetic biology tools. Complex pathways can be constructed in different conformations by using DNA assembly tools. Large DNA fragments are constructed by using one step assembly tools like sequence and ligation independent cloning (SLIC), Gibson isothermal assembly, ePath Brick vectors and Circular Polymerase Extension Cloning (Gibson et al., 2009; Quan and Tian, 2011; Jeong et al., 2012; Xu et al., 2012; He et al., 2015). Xu and colleagues increased the production of flavanone naringenin by assembling transcription components and genes of the pathway in a module and also by fine-tuning the gene expression using the ePathBrick vector system. This is a system that allows rapid cloning of all possible gene conformations by using the isocaudamer restriction sites. Two multigene pathways were present in this pseudooperon gene structure consisting of the genes for naringenin biosynthesis and for biosynthesis of malonyl CoA (acetyl CoA carboxylase complex or ACC). 80 mg/L naringenin production was achieved by using this gene construct (Xu et al., 2012). Up to 911 mg/L titer of (+) catechin was produced from 1 g/L eriodictyol in batch culture by using the same ePathBrick vector system (Zhao et al., 2015).

Recombinant proteins and metabolic pathways can be expressed by using chromosomal integration instead of plasmids. Contrary to common perception, high expression levels of recombinant genes, ranging from 25 to 250% can be achieved through chromosomal integration. In one recent example, the protein expression level at four genomic loci on the *E. coli* chromosome was measured by integrating fluorescent reporter protein mCherry into each loci. The result demonstrated that the location of the chromosomal integration significantly affected protein expression; this was further shown by integrating a gene encoding for TAL at the same loci resulting in various production titers of trans–cinnamic acid (Englaender et al., 2017).

As stated above, low intracellular concentration of malonyl CoA is a major limiting factor for the production of flavonoids in recombinant *E. coli*. The identification of appropriate genetic targets for desired manipulations in the metabolic make up of the microbes is a difficult task (Gerosa and Sauer, 2011; Lee et al., 2012). Commonly used systematic approaches are based on stoichiometric models of host cells and various algorithms have been developed and applied in the past several years to that effect. In the case of malonyl CoA and flavonoid production, in an early example an algorithm based on the genetic algorithm and termed cipher of evolutionary design (CiED) was developed by Fowler et al. (2009); it allowed the identification of knock-outs that resulted in increased malonyl CoA biosynthesis by deleting a number of genes that were predicted to negatively influence the intracellular concentration of malonyl-CoA in *E. coli*. CiED can also optimize the host strain by predicting the genes to be overexpressed like acetyl CoA carboxylase genes, genes for acetate assimilation, for the coexpression of plant derived flavanones and for the biosynthesis of coenzymeA. CiED aided engineering produced an optimized strain by selective deletions and overexpressions which increased naringenin production by over 660% and eriodictyol by 420%. Later on, in another example, a customized version of a FBA algorithm called OptForce (Ranganathan et al., 2010) was used to predict minimal sets of genetic interventions, again for optimizing flavanone production in *E. coli*. One of the predicted strains exhibited a 4-fold increase in the levels of intracellular malonyl CoA compared to the wild type BL21 *E. coli* strain and resulted in the production of 474 mg/L of naringenin (Xu et al., 2011).

As already mentioned, transporters are a major mechanism for relieving toxicity and are well studied in the context of antibiotic tolerance and a vast range of compounds are exported by them. They specifically have emerged as a powerful category of proteins that provide tolerance and often improve production titers; however, they are difficult targets for cellular expression (Mukhopadhyay, 2015). Tolerance engineering refers to the specific area of host optimization to overcome the sensitivity to the final product by engineering tolerance mechanisms in the host. Enhanced export and weakened uptake process could relieve the cellular toxicity as well as increase the final product titer (Ling et al., 2014; Peabody et al., 2014). For example, the tolerance to isoprenoids was increased by the overexpression of some heterologous efflux pumps, thereby increasing the productivity titer (Dunlop et al., 2017). Similarly, resveratrol production was increased by 2.44-fold in an engineered yeast by heterologous expression of a low affinity and high capacity arabinose transporter AraE from *E. coli* (Wang et al., 2011a).

## Anthocyanins

Anthocyanins are the water-soluble colored pigments that are present in terrestrial plants. Anthocyanins are also responsible for providing blue, purple and red colors to many fruits and flowers. In the plant kingdom, being a member of the flavonoid group of polyphenols, anthocyanins are important chemicals as pigments, antimicrobials and antioxidants. Visible and ultraviolet (UV) spectra are strongly absorbed by anthocyanins due to their specific polyphenol structure (Giusti and Wrolstad, 2001) and when applied externally, anthocyanins play a role in the protection of human skin from aging and damage induced by UV rays (Chan et al., 2010) such as inflammation and oxidative damage in the dermis, epidermis and adnexal organs of the skin. Such discoveries have resulted in the increasing application of anthocyanins in cosmetics and skin care products (Rojo et al., 2013). By blocking the action of interleukin-1β, tumor necrosis factor α, and nuclear factor-κβ, anthocyanins are also reported to help in the suppression of neuroinflammation, neurodegradation and brain aging (Tsuda, 2012). Moreover, the therapeutic efficacies of anthocyanins have been further demonstrated by studies conducted in animal models and humans (Burton-Freeman et al., 2010). Further, anthocyanins have also been reported to be effective in the prevention of neurodegenerative diseases, diabetes, cardiovascular diseases, obesity and cancer (de Pascual-Teresa et al., 2010; Tsuda, 2012). Due to the diverse colors and nutritional properties of anthocyanins, they are widely used as food colorants. Four anthocyanin-based colorants are exempt from FDA certification in the US. In industry, because of their improved color stability, acylated anthocyanins are also commonly applied (Giusti and Wrolstad, 2003). In order to replace the complicated, toxic, and costly transition metal coordination complexes, anthocyanins are also exploited in dye-sensitized solar cells (DSSCs) as sensitizers. This is done for converting solar energy to electricity with slightly lower, yet acceptable efficiencies than the traditional silicon solar cells (Calogero et al., 2012; Ramamoorthy et al., 2016).

**FIGURE 4:**
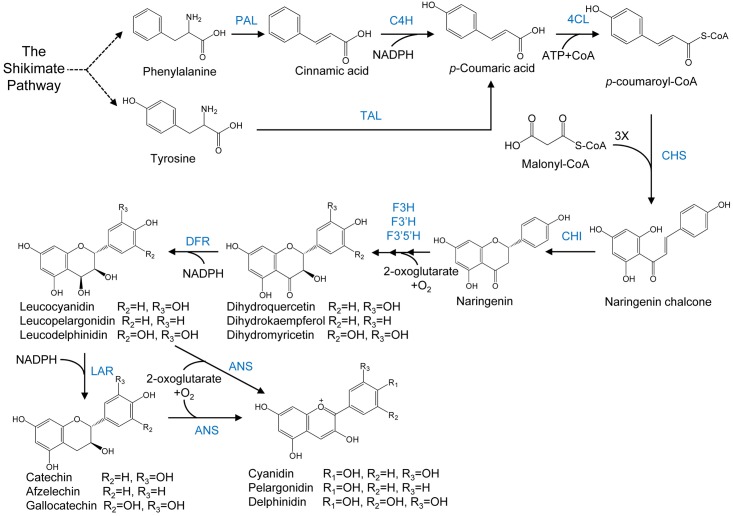
Anthocyanin biosynthesis. The general phenylpropanoid pathway is catalysed by phenylalanine ammonia lyase (PAL), cinnamate 4-hydroxylase (C4H) and 4-coumaryol CoA ligase (4CL). Enzymes involved in flavonoid biosynthesis are chalcone synthase (CHS), chalconeisomerise (CHI), flavanone 3-hydroxylase (F3H), flavanone 3′5′- hydroxylase, (F3′5′H) and flavanone 30-hydroxylase (F3′H). Anthocyanidins are synthesized by dihydroflavonol 4-reductase (DFR) and anthocyanidin synthase (ANS). Anthocyanins can also be synthesized from flavan-3-ol derived from leucoanthocyanidin by leucoanthocyanidin reductase (LAR).

Extraction and purification from flowers, fruits and other tissues of plants are among the traditional means of producing anthocyanins (Corrales et al., 2008; Aberoumand, 2011; Ananga et al., 2013; Mora-Pale et al., 2014). An example of a commercial process for the production of anthocyanins through plant extraction is the use of red cabbage for the extraction of cyanidin 3-*O-*glucoside extract by Colarome, a chemical company in Quebec, Canada. A large number of plant species have been reported in the literature for the production of anthocyanins using callus and suspension cultures, including *Vitis vinifera*, *Perilla frutescens*, *Sorghum bicolor Moench*, *Daucus carota*, *Centaurea cyanus*, *Fragaria* sp., *Fagopyrum esculentum*, *Fagoprum tataricum*, *Aralia cordata*, *Catharanthus roseus*, *Petunia hybrida*, *Euphorbia milii*, *Populus deltoides*, *Oxalis reclinata*, *Vaccinium pahalae*, *Hyoscyamus muticus L.*, Maize, *Eggplant hypocotyl*, *Ajuga reptans*, *Sweet potato*, *Rosmarinus officinalis*, *Malus sp.*, *Chrysanthemum coronarium*, *Hibiscus sabdariffa*, *Arabidopsis thaliana*, *Ipomoea batatas*, *Brassicaceae*, *Glycine max L. Merr.*, *Carnelia sp.*, *Glehnia littoralis*, *Taraxacum officinale*, *Allium cepa*, *Capsicum frutescens*, *Ajuga pyramidalis Metallica Crispa*, *Oryza sativa L.*, and *Hydrilla verticillata* (Zhang and Furusaki, 1999; Rao and Ravishankar, 2002). Production of anthocyanins through plant extraction is neither stable nor sustainable because production of anthocyanins in plants fluctuates in response to seasonal as well as environmental conditions. Production of anthocyanins in microbes is an alternative to this problem as this has displayed good potential in the biosynthesis of natural compounds derived from plants (Staniek et al., 2014; Pandey et al., 2016). Anthocyanin biosynthesis proceeds via the phenylpropanoid pathway, as shown in **Figure 4**.

### Metabolic Engineering of Anthocyanin Biosynthesis in Microbes

As already discussed in previous sections of this manuscript, biosynthesis of natural flavonoids in microbes dates back to 2003 and since then, various flavonoid compounds from flavanones to the more complicated anthocyanins, have been reported to be synthesized in engineered microorganisms (Wang et al., 2011a; Pandey et al., 2016). In a first attempt toward recombinant anthocyanin biosynthesis, the genes of flavanone 3-hydroxylase (F3H) and ANS from *Malus domestica*, DFR from *Anthurium andraeanum*, and flavonoid 3-*O-*glucosyltransferase (F3GT) from *Petunia hybrida* were reported to be cloned and expressed in *E. coli*. The resulting strain produced 6.0 μg/L of cyanidin 3-*O*-glucoside and 5.6 μg/L pelargonidin 3-*O*-glucoside by using naringenin and eriodictyol as precursors (Yan et al., 2005).

In order to improve these low titers, artificial enzyme clusters were created by translationally fusing multiple enzymes in successive steps. A higher titer of cyanidin 3-*O*-glucoside was achieved by fusing F3GT from *Arabidopsis thaliana* with the N-terminus of ANS from *Petunia hybrida* with a pentapeptide linker, with the chimeric enzyme in comparison to the uncoupled ANS and F3GT. Presumably, the successive biochemical reactions were catalyzed more efficiently by the chimeric enzyme complex as compared to the independent enzymes because of their faster conversion of the unstable intermediate cyanidin (Yan et al., 2008). Such enzyme clusters were further investigated in the case of flavan-3-ol production in recombinant *E. coli* (Zhao et al., 2015).

For the biosynthesis of anthocyanins in an efficient way, sufficient supply of UDP-glucose is indispensible. This was achieved by overexpression of UDP-glucose biosynthetic genes (*pyrE*, *pyrR*, *cmk*, *ndk*, *pgm*, *galU*) along with partial inhibition of the UDP-glucose degradation pathways. Production of cyanidin 3-*O*-glucoside was increased by 20-fold due to these modifictions (Leonard et al., 2008). In another study, a 57.8% increase in the production of cyanidin 3-*O*-glucoside by the overexpression of intracellular genes *pgm* and *galU* along with the expression of ANS and 3GT was reported (Yan et al., 2008).

For the efficient production of anthocyanins, various other factors need to be optimized including pH, induction time-point, temperature, substrate feeding and amount of dissolved oxygen. It was reported that in engineered *E. coli*, induction at the stationary phase was optimal for cyanidin 3-*O*-glucoside production. Along with this, pulsing of (+)-catechin and glucose resulted in improved production of anthocyanins. In addition, over expression of YadH, a cyanidin 3-*O*-glucoside-associated efflux pump resulted in 15% more production of anthocyanins. Production titer of cyanidin 3-*O*-glucoside was further improved by deletion of another efflux pump TolC that is probably responsible for the secretion of the substrate catechin (Lim et al., 2015).

Further more, Jones et al. (2017) described the complete biosynthesis of anthocyanins using *E. coli* polycultures. This work was a continuation of a previous work that demonstrated the use of a two-strain co-culture for the efficient production of flavan-3-ols from precursor phenylpropanoic acids, an approach that resulted in a 58-fold improvement in final production titers compared to a monoculture strategy (Jones et al., 2016). It is the first report on engineering complex microbial biosynthesis of an anthocyanin plant natural product, starting from sugar. Accomplishment of this was achieved by the development of a synthetic, 4-strain *E. coli* polyculture that collectively expressed 15 exogenous or modified pathway enzymes from diverse plants as well as other microbes. The functional expression and connection of lengthy pathways was enabled by this synthetic consortium-based approach apart from the effective management of the accompanying metabolic burden. The utilization of polyculture strategy afforded milligram-per-liter production titers.

## Curcuminoids

A major component present in turmeric is curcumin, which is a member of the curcuminoids class of plant polyphenols. Curcuminoids belong to the polyphenolic family, and have carbon skeleton of diarylheptanoids (C6-C7-C6) providing yellow color to turmeric. They are isolated from the rhizome of turmeric (*C. longa* Linn.) where they provide yellow color to turmeric (Srinivasan, 1953; Jayaprakasha et al., 2005; Palve and Nayak, 2012). In addition to curcumin, bisdemethoxycurcumin and demethoxycurcumin are among the other curcuminoids present in such mixtures. Many species of *Curcuma* (like *phaeocaulis, aromatic, mangga, xanthorrhiza*) produce curcumin, demethoxycurcumin and bisdemethoxycurcumin (Mohamad et al., 2005; Tohda et al., 2006; Lobo et al., 2009; Malek et al., 2011). In these plants, the contribution of circumnoids to the dry weight of rhizome is influenced by cultivar and ranges from 2 to 4% (Ravindran et al., 2007). *Zingiber cassumunar* also produces some curcuminoids, such as cassumunin and cassumunarin (Masuda et al., 1995). Currently, the powdered dry rhizome of *C. longa* is used for the isolation of commercial-grade curcumin and contains a mixture of curcumins (∼77%), demethoxycurcumin (∼8%) and bisdemethoxycurcumin (∼5%) (Goel et al., 2008).

Curcumin demonstrates a number of beneficial properties in human health. It possesses the ability to suppress acute and chronic inflammation (Shishodia et al., 2005; Jiao et al., 2006). Curcumin is reported to inhibit cell proliferation and metastasis at a molecular level. Apoptosis is also induced by curcumin through modulation of receptors (e.g., epidermal growth factor receptor, human epidermal growth factor receptor -, IL-8R and Fas-R), pro-inflammatory factors [e.g., interleukin (IL)-1, IL-1β, IL-12, tumor necrosis factor and interferon], growth factors (e.g., epidermal growth factor, hepatic growth factor and platelet-derived), and several transcription factors (Ravindran et al., 2009; Bar-Sela et al., 2010). In addition, curcumin is reported to enhance wound healing and provides protection against cataract formation, liver injury, fibrosis and pulmonary toxicity (Aggarwal, 2010; Shin et al., 2011). Although curcumin has the potential to prevent and cure many diseases, their bioavailability is limited. Curcuma species are the only natural sources of curcuminoids but plants of the Zingiberales order have been reported to possess curcuminoids and related compounds (Katsuyama et al., 2008).

### Curcuminoids Biosynthesis in Plants

**FIGURE 5:**
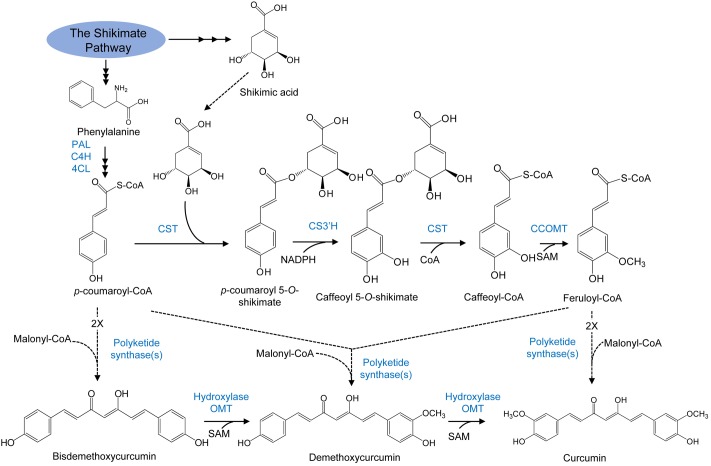
Curcuminoid biosynthesis pathway in plants. Enzymes are as follows: PAL, phenylalanine ammonia lyase; C4H, cinnamate 4-hydroxylase; 4CL, 4-coumarate: CoA ligase; CST, *p*-coumaroyl shikimate transferase; CS3′H, *p*-coumaroyl 5-*O*-shikimate 3′-hydroxylase; OMT, *O*-methyltransferase; SAM, *S*-adenosyl-L-methionine; CCOMT, caffeoyl-CoA O-methyltransferase (adapted from Ramirez-Ahumada et al., 2006).

Polyketide synthase catalyzes the synthesis of curcuminoids by condensing one molecule of malonyl CoA with two molecules of *p*-coumaroyl-CoA (**Figure 5**) (Brand et al., 2006). Two sequential rounds of hydroxylation, followed by *O*-methylation reactions transform the resulting bisdemethoxycurcumin into curcumin through demethoxycurcumin. The enzyme curcuminoid synthase may also use the CoA esters of *p*-coumaric acid and ferulic acid as substrates. The central pathway could be operative in this case and the hydroxylation as well as the *O*-methylation reactions resulting in the formation of methoxyl functional groups in curcumin would occur via the same reactions like those in the phenylpropanoid pathway.

### Metabolic Engineering of Curcuminoids Biosynthesis in Microbes

Recombinant *E. coli* strains have been developed for the production of curcuminoids. In the work by Katsuyama and co-workers, three different sources were used for the isolation of genes namely PAL, 4CL and CUS (curcuminoid synthase) needed for curcuminoid biosynthesis. The genes encoding for these enzymes were derived from *Rhodotorula rubra*, *Lithospermum erythrorhizon* and *Oryza sativa* respectively. The engineered strain produced relatively modest titer of curcuminoids (113 mg/L of curcumin) (Katsuyama et al., 2008). Similarly, Morita et al. (2010) showed that it is easy to artificially biosynthezise curcuminoids by using CUS than the DCS (diketide Co-A synthase)/CURS (curcumin synthase) system. In another study, precursor directed biosynthesis of curcumin analogs in *E. coli* was also reported. In this study, 17 unnatural curcuminoids were produced when the *E. coli* cells engineered for the production of curcuminoids were supplied with carboxylate precursors exogeneously. Further, asymmetric curcuminoids were also produced by adding two different precursors simultaneously. It was concluded that modification of the culture conditions and substrate specificity of CUS and 4CL should be useful for improvement of the yield of unnatural curcuminoids (Katsuyama et al., 2010). In another study, Rodrigues et al. (2015) reported the production of natural curcuminoids through caffeic acid by engineering a synthetic pathway in *E. coli.* In this study, *E. coli* strain was engineered with genes for diketide-CoA synthase (DCS) and curcumin synthase (CURS1), isolated from *C. longa* and the gene for 4-coumaroyl-CoA ligase isolated from *Arabidopsis thaliana.* Joana et al. (2017), produced curcumin in *E. coli* through heat induction using the *ibpA* and *dnaK* heat shock promoters. 17 μM curcumin, was produced using diketide-CoA synthase (DCS) and curcumin synthase 1 (CURS1) from *C. longa* and 4-coumarate-CoA ligase (4CL1) from *Arabidopsis thaliana* (Joana et al., 2017).

In another recent study, a recombinant *E. coli* strain was constructed by cloning either *PAL* from *A. thaliana* or *TAL* from *Saccharothrix espanaensis*, together with *4CL* from *O. sativa* and *CUS* also from *O. sativa.* The resulting strains were able to produce a number of different curcuminoids, including bisdemethoxycurcumin, dicinnamoylmethane, and cinnamoyl-*p-*coumaroylmethane at concentrations of 4.63 mg/L, 6.95 mg/L and 1.11 mg/L respectively from tyrosine (Kim et al., 2017).

## Future Perspectives

Various plant secondary metabolites like flavonoids, anthocyanins and curcuminoids, are produced by plants and have shown to have numerous health benefits. They have also been used in functional foods, cosmetics and nutraceuticals. But plants produce them only in limited amounts and also under some specific environmental conditions or under some stress. Metabolic engineering and synthetic biology approaches have generated microbial cell factories that can allow the large-scale production of these pharmaceutically and nutraceutically important metabolites in an environmentally friendly and efficient way. One advantage of recombinant microorganisms is their ability to produce relatively pure compounds that do not require extensive downstream processing. In addition, they offer the ability to produce novel, non-natural derivatives with potentially better properties.

There is little doubt that advances in synthetic biology have significantly accelerated our ability to optimize or create cell factories. However, in order for the recombinant microorganisms to provide competitive processes for the production of polyphenolic compounds, titers approaching the gram per liter scale are necessary. For increasing the efficiency of microbial cell factories for yield improvement, it is necessary to have a comprehensive knowledge of the intracellular make up of the host cell, i.e., its entire genome, transcriptome, proteome and metabolome. Moreover tools of synthetic biology and computational biology can identify genetic manipulation targets so as to bring the desired changes in strain which increases its production capacity. The challenge before scientists is to improve the yield of beneficial metabolites. Metabolic limitations and bottlenecks have to be identified, and should be considered from the perspective of the whole organism.

## Author Contributions

All authors listed have made a substantial, direct and intellectual contribution to the work, and approved it for publication.

## Conflict of Interest Statement

The authors declare that the research was conducted in the absence of any commercial or financial relationships that could be construed as a potential conflict of interest.
